# Adiponectin, diabetes and ischemic heart failure: a challenging relationship

**DOI:** 10.1186/1475-2840-11-151

**Published:** 2012-12-18

**Authors:** Samuele Baldasseroni, Alessandro Antenore, Claudia Di Serio, Francesco Orso, Giuseppe Lonetto, Nadia Bartoli, Alice Foschini, Andrea Marella, Alessandra Pratesi, Salvatore Scarantino, Stefano Fumagalli, Matteo Monami, Edoardo Mannucci, Niccolò Marchionni, Francesca Tarantini

**Affiliations:** 1 Department of Critical Care Medicine and Surgery, Geriatric Medicine Unit, University of Florence, Florence, Italy; 2 Department of Heart and Vessels, Geriatric Cardiology and Medicine Unit, University of Florence and Azienda Ospedaliero-Universitaria Careggi [AOUC], Viale Morgagni 85, 50134, Florence, Italy

**Keywords:** Adiponectin, Diabetes, Coronary artery disease, Heart failure

## Abstract

**Background:**

Several peptides, named adipokines, are produced by the adipose tissue. Among those, adiponectin (AD) is the most abundant. AD promotes peripheral insulin sensitivity, inhibits liver gluconeogenesis and displays anti-atherogenic and anti-inflammatory properties. Lower levels of AD are related to a higher risk of myocardial infarction and a worse prognosis in patients with coronary artery disease. However, despite a favorable clinical profile, AD increases in relation to worsening heart failure (HF); in this context, higher adiponectinemia is reliably related to poor prognosis. There is still little knowledge about how certain metabolic conditions, such as diabetes mellitus, modulate the relationship between AD and HF.

We evaluated the level of adiponectin in patients with ischemic HF, with and without type 2 diabetes, to elucidate whether the metabolic syndrome was able to influence the relationship between AD and HF.

**Results:**

We demonstrated that AD rises in patients with advanced HF, but to a lesser extent in diabetics than in non-diabetics. Diabetic patients with reduced systolic performance orchestrated a slower rise of AD which began only in face of overt HF. The different behavior of AD in the presence of diabetes was not entirely explained by differences in body mass index. In addition, NT-proBNP, the second strongest predictor of AD, did not differ significantly between diabetic and non-diabetic patients. These data indicate that some other mechanisms are involved in the regulation of AD in patients with type 2 diabetes and coronary artery disease.

**Conclusions:**

AD rises across chronic heart failure stages but this phenomenon is less evident in type 2 diabetic patients. In the presence of diabetes, the progressive increase of AD in relation to the severity of LV dysfunction is hampered and becomes evident only in overt HF.

## Background

The adipose tissue should not be regarded as a simple site of lipid storage [1]; indeed it is able to secrete several peptides with hormonal properties that are involved in energy homeostasis as well as modulation of inflammation and regulation of many immunological mechanisms [2]. This heterogeneous group of hormones is named adipokines. Among these, adiponectin (AD) is by far the most abundant protein secreted by the adipose tissue [3]. Although it is produced almost exclusively by adipocytes, plasma levels of AD are found to be inversely correlated to visceral adiposity and body mass index (BMI) [3]. AD promotes peripheral insulin sensitivity [4] and inhibition of liver gluconeogenesis [5]. As a matter of fact, hypoadiponectinemia is known to be the molecular link between obesity and insulin-resistance, at the base of metabolic syndrome [2]. AD is able to predict diabetes onset and diabetic patients always show lower plasma levels of AD compared to the general population [6]. In addition, it has been reported that plasma level of adiponectin in diabetic subjects with foot ulceration was lower than in diabetic subjects without diabetic foot [7], suggesting that hypoadiponectinemia may be related to the microvascular complications of diabetes.

Beside of its beneficial role in the glycometabolic pathway, AD shows several favorable properties as an anti-atherogenic and anti-inflammatory agent [8]. Several studies report an AD mediated inhibition of many peptides and cytokines involved in the progression of atherosclerosis [9]. At the same time, AD seems to counteract the transition from stable atheromatic plaques to vulnerable ones [10]. Given these properties, it is not unexpected that hypoadiponectinemia is related to coronary artery disease (CAD) [11]. In fact plasma, levels of AD are able to predict not only the risk of myocardial infarction but also the prognosis and clinical course of CAD [10].

Because of all the beneficial effects on insulin resistance and atherosclerosis, it came as a surprise the observation that plasma levels of AD were elevated in chronic heart failure (HF) [12]. In fact, AD seems to increase in relation to worsening HF [13] and it is reliably related to poor prognosis [14]. Three main hypotheses have been proposed to explain this apparent paradox. The first one suggests that cachexia related to HF is responsible for the upsurge of AD [15]. A second hypothesis relates to the possibility of an adiponectin resistance: indeed, a lower density of AD receptors has been detected on musculoskeletal cells from HF patients [16]. The last suggestion came from an elegant study by Tsukamoto and coll. that showed how NT-proBNP is able to induce AD secretion from adipocytes. Furthermore, they found that injection of NT-proBNP in humans caused an increase in AD when compared to saline solution [17]. Based on an overall analysis of all data available, Park and coll. recently speculated that although high adiponectin levels may confer cardioprotection in the acute setting, i.e. during acute myocardial infarction, it may contribute in a permissive way to adverse remodeling in the post-acute period, accelerating the transition to heart failure [18].

Several factors are known to predict adiponectinemia; among those NT-proBNP and BMI are the most relevant [19]. However, little or nothing is known about how certain metabolic conditions, such as diabetes mellitus (DM), modulate the correlation between AD and HF.

Our aims were i) to evaluate plasma levels of AD in patients affected by ischemic HF with and without type 2 DM, ii) to analyze the correlation between adiponectinemia and ACC/AHA stage of chronic HF, and iii) to elucidate whether the presence of DM was able to influence the relationship between HF and AD levels.

## Methods

### Study subjects

We enrolled 150 consecutive outpatients referred to the Geriatric Cardiology and Medicine Unit of Careggi Academic Hospital (Florence, Italy) for CAD between January 1^st^, 2008 and December 31^st^, 2010. All patients were Caucasians and met the following inclusion criteria: written informed consent, CAD documented by at least one ≥ 75% stenosis of a major epicardial branch at coronary angiography, no hospitalization for worsening HF during the last two months. Those with type 2 diabetes were classified according to ADA criteria [20]. The study was approved by an institutional review committee.

### Clinical and instrumental data

**Figure 1:**
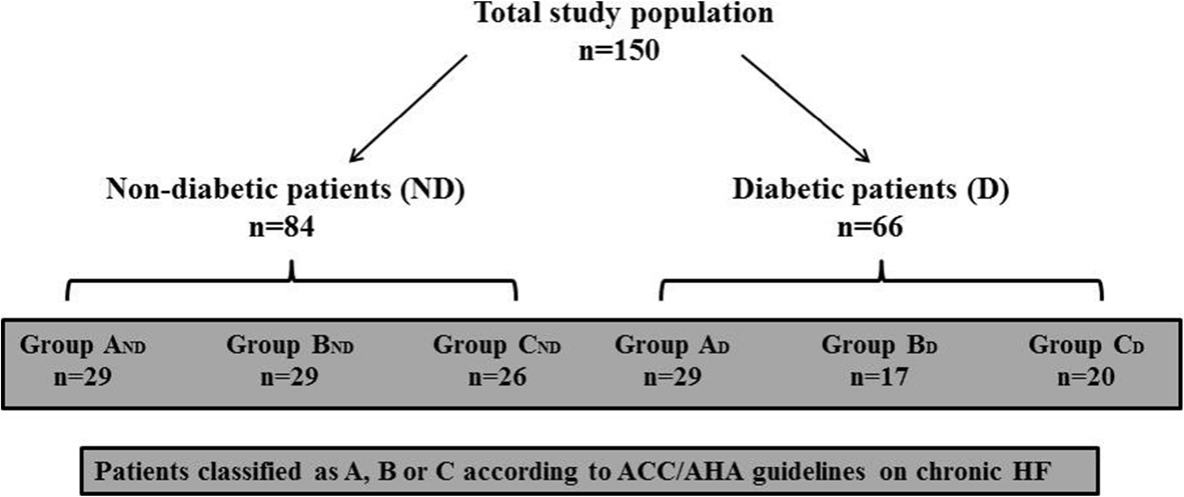
Flow chart of the study.

We collected a thorough clinical history, including symptoms needed to define New York Heart Association (NYHA) class. Patients underwent physical examination with measurement of height and weight to calculate BMI, 12-lead electrocardiogram, and 6-min walking test (6MWT) [21]. A standard echocardiographic study was carried out following the American Society of Echocardiography recommendations [22] using a 4-chamber apical view to calculate left ventricular ejection fraction (LVEF), according to the Simpson’s formula. Heart failure was considered to be overt in the presence of positive European Society of Cardiology criteria [23], coupled with a Boston score ≥5 [24]. As reported in the flow chart of Figure 1, the study population was divided into two groups: the diabetes (D) group which included patients with diagnosis of type 2 DM, and the non-diabetes (ND) group.

Combining LVEF with ESC and Boston criteria and according to ACC/AHA guidelines for chronic HF [25] each group was further divided into three subgroups as follows: group A including patients with LVEF ≥55%, no overt HF; group B, LVEF <40%, no overt HF and group C, LVEF <40%, overt HF. Patients with LVEF in the 40-54% range were excluded from the study in order to select subjects with markedly different LV systolic function. Subgroups were marked as follows: A_ND_, B_ND_ and C_ND_, including patients without diabetes, and A_D_, B_D_ and C_D_ which comprised patients with type 2 DM (Figure 1).

The level of non-cardiac comorbidity was measured with the Greenfield’s Index of Disease Severity (IDS) score [26], whereas the presence of depressive symptoms was evaluated with the 30-item form of the Geriatric Depression Scale (GDS) [27].

### Biochemical data

Venous blood samples were drawn in the fasting state to detect glycaemia, hemoglobin (Hb) and glycated Hb (HbA1c) which was analyzed by high liquid pressure chromatography (Menarini Diagnostics, Florence, Italy; upper normal limit 5.9%). Glomerular filtration rate (eGFR) was estimated according to the Modification of Diet in Renal Disease (MDRD) Study method [28]. Plasma level of AD was determined, in duplicate, by ELISA (Linco Research, Missouri, USA); NT-proBNP was measured with a chemiluminescent immunoassay kit (Roche Diagnostic Laboratory, Indianapolis, IN, USA) on an Elecsys 2010 analyzer. All other biohumoral parameters are reported in Table 1.

**Table 1:** Characteristics of the study population

**Variable**	**Total n=150**	**ND Group n=84**	**D Group n=66**	**p value**
**Age (years)**	68.8±10.8	67.6±12.6	70.2±7.6	0.143
**Female**	13%	14%	12%	0.699
**BMI (Kg/m**^**2**^**)**	26.7±4	25.2±3.4	28.8±4.2	0.001
**History of angina**	42%	36%	52%	0.060
**Previous ACS**	79.6%	82%	79%	0.500
**Previous PTCA**	75%	73.6%	80.1%	0.307
**Previous CABG**	27.6%	27.3%	28.8%	0.921
**Atrial fibrillation**	17.8%	13.1%	24.2%	0.084
**Hypertension**	69.7%	66.7%	75.8%	0.267
**Dyslipidemia**	69.7%	70.2%	71.2	0.986
**Smoking**	63%	51.2%	80%	0.001
**COPD**	12.5%	11.9%	13.6%	0.773
**NYHA class III-IV**	19.4%	15.5%	13.6%	0.982
**Boston score**	2.5±3.4	2.7±3.7	2.2±3.1	0.345
**6-min walk test (m)**	430.3±170.3	458±180.9	395.8±150.8	0.031
**Index Disease Severity (IDS) score**	3.7±2.5	2.7±2.1	4.9±2.3	0.345
**GDS score**	7.4±5.2	7.1±5.2	7.7±5.3	0.493
**Third heart sound**	5%	7%	3%	0.259
**Peripheral edema**	16.9%	10%	24%	0.016
**Jugular vein distention**	11.9%	11.9%	12.1%	0.989
**Pericardial fat (mm)**	7.9±3.1	7.2±2.8	8.4±3.4	0.041
**Myocardial mass**	129.6±35.3	130.2±33,9	128.9±37.3	0.835
**Ejection fraction (%)**	43.9±15	41.3±14.5	47.3±15.6	0.017
**Trans-tricuspid gradient (mmHg)**	19.9±13.6	21±14	18.5±12.9	0.263
**Glycaemia (mg/dl)**	118.5±45	94.5±11.9	149.7±52.7	0.001
**Hemoglobin (g/dl)**	13.6±1.5	13.7±1.5	13.4±1.5	0.202
**HbA1c (%)**	6.64±1.27	5.9±0.3	7.6±1.4	0.001
**eGFR (ml/min)**	71.3±25.1	67.8±21.2	75.8±28.8	0.058
**Total cholesterol (mg/dl)**	170.1±41.8	171.9±39.6	167.9±44.7	0.576
**HDL (mg/dl)**	43.8±11.5	43.1±10.3	44.8±13	0.363
**ESR (mm/h)**	33±25.2	36.1±27.4	29.1±21.6	0.101
**TSH (mU/l)**	2.1±3.7	2.9±4.2	2.2±3	0.786
**Albumin (g/dl)**	5.1±0.8	3.9±0.4	3.7±0.4	0.147
**NT-proBNP (ng/ml)**	2101.9±4246.6	2066.8±3243.2	2071.4±5300.1	0.995
**ACE/ARB**	89.5%	88%	93.9%	0.222
**Beta-blockers**	82.2%	86.9%	78.8%	0.185
**Digitalis**	19.7%	19%	21.2%	0.742
**Aldosterone blockers**	25%	33.3%	15.2%	0.011
**Loop diuretics**	53.3%	52.4%	56.1%	0.654
**Nitrates**	29.6%	31%	28.8%	0.774
**Statins**	78.3%	85.7%	71.2%	0.029
**Warfarin**	15.1%	15.5%	15.2%	0.956
**Aspirin**	84.2%	88%	81.8%	0.281
**Clopidogrel**	40.1%	50%	28.8%	0.009

Abbreviations: BMI, Body Mass Index; ACS, Acute Coronary Syndrome; PTCA, Percutaneous Transluminal Coronary Angioplasty; CABG, Coronary Artery Bypass Graft; COPD, Chronic Obstructive Pulmonary Disease; GDS, Geriatric Depression Scale; HbA1c, Glycated Hemoglobin; eGFR, estimated Glomerular Filtration Rate; ESR, Erythrocyte Sedimentation Rate; TSH, Thyroid Stimulating Hormone; ACE/ARB, Angiotensin Converting Enzyme/ACE Receptor Blockers.

### Statistical analysis

Data were analyzed using SPSS^®^ software (version 17) and are presented as mean ± SD or median (quartiles) - depending on their normal or non-normal distribution - for continuous variables, and as number (%) for categorical ones. Categorical and continuous variables were compared between the two groups of patients (ND and D) using the Pearson *T*-test and the chi-square test, respectively. The difference in AD plasma levels across the groups was analyzed as follows: first we used univariate ANOVA model to test the difference among the three ACC/AHA HF stage subgroups, separately in the ND and D group; then we compared the level of AD by coupling the matching stage of ND and D groups. Finally, BMI, NT-proBNP and HDL cholesterol were introduced in the univariate ANOVA models as covariates; this choice was supported by the fact that these three variables have proven to be the strongest predictors of AD, as previously reported by us [19] and others [3,13]. In the last step, we tested different multivariate backward stepwise linear regression models with AD as dependent variable and age, gender, ACC/AHA HF stage, BMI, NT-proBNP, HDL cholesterol and DM as independent variables, to test their independent predictive value.

## Results

### Comparison between ND and D groups

The main features of the study population and the main differences between the two groups (ND and D) are summarized in Table 1. Briefly, a history of angina and the presence of atrial fibrillation were slightly more prevalent among diabetics, although not significant. As expected glycaemia, HbA1c and BMI were significantly higher in group D patients, as well as smoking. We also noticed a worse performance at the 6MWT among diabetics which were also more likely to display peripheral edema despite a better mean LVEF. In accordance to the literature [29], pericardial fat tissue was thicker in diabetics because of their higher BMI. With the exception of clopidogrel and aldosterone blockers, drugs reported to be used were similar in the two groups; unexpectedly, statins were more frequently prescribed in the ND group. Among diabetics, 14 patients (21.2%) were treated with diet alone, 14 (21.2%) were prescribed metformin and 12 (18.2%) a combination of two oral anti-diabetic drugs. Twenty-six patients (39.4%) were treated with insulin.

### Adiponectin

**Figure 2:**
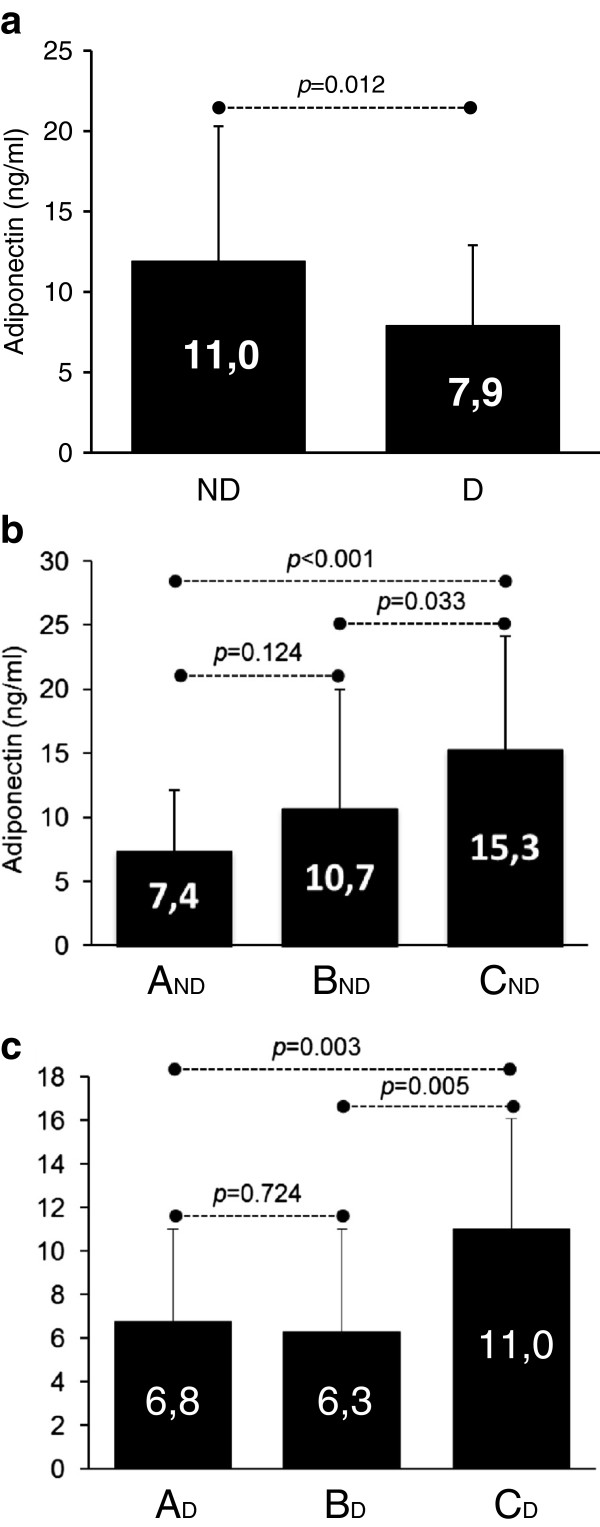
Comparison of adiponectin levels in Non-Diabetic (ND) vs. Diabetic groups (a), and across different clinical stages (A, B and C) of HF, separately in ND and D (b and c).

**Figure 3:**
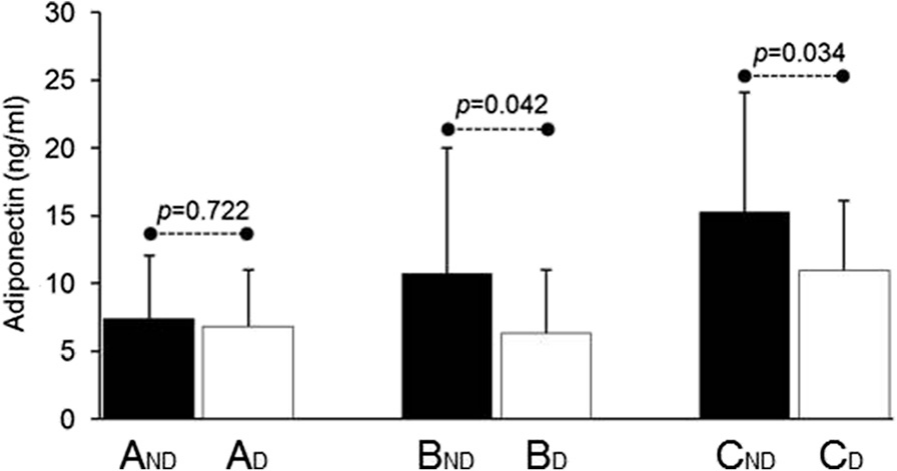
Comparison of adiponectin levels in subjects belonging to the same clinical stage of HF (A, B and C) but with different metabolic profile (ND, Non-Diabetics vs. D, Diabetics).

Mean AD level was 9.7±7.3 ng/ml with a significantly higher level in the ND compared to the D group (11.0±8.4 vs 7.9±5.0 ng/ml, p=0.012) (Figure 2A). As shown in Figure 2B, there was a slight, but not significant, increase of AD in group B_ND_ compared to A_ND_. A significant rise was observed in group C_ND_ compared to the other two (p=0.001 and p=0.003, respectively). Among diabetic subjects (Figure 2C) no difference was found between A_D_ and B_D_ (6.8±4.2 ng/ml vs 6.3±4.7 ng/ml, p=0.742), while C_D_ showed a significant increase (11.0±5.1 ng/ml) compared to both A_D_ (p=0.003) and B_D_ (p=0.005). We finally matched subjects belonging to the same clinical stage of HF (A, B, C) but with different metabolic profile (ND vs. D) (Figure 3): no difference was found among diabetic and non diabetic patients in group A (A_ND_ 7.4±4.7 ng/ml vs. A_D_ 6.8±4.2 ng/ml, p=0.722); AD levels became significantly different in group B (B_ND_ 10.7±9.3 ng/ml vs. B_D_ 6.3±4.7 ng/ml, p=0.042) and even more divergent in group C (C_ND_ 15.3±8.8 ng/ml vs. C_D_ 11.0±5.1 ng/ml, p=0.034).

When the analysis of variance of AD in different clinical stages of HF was adjusted for BMI, HDL cholesterol and NT-proBNP - the main cofactors known to predict AD plasma levels – the difference in AD still remained significant in the total population and in the ND group (Table 2). Conversely, in the diabetic group the difference after covariate adjustment was not significant.

**Table 2:** Analysis of variance of adiponectin plasma levels across the three subgroups (A, B and C)

**Model**	**Unadjusted**		Adjusted*	
	**F**	***p***	**F**	***p***
**Total poulation**	10.9	<0.001	5.4	0.005
**ND group**	6.7	0.002	6.1	0.004
**D group**	6.0	0.004	1.4	0.250

*adjusted for BMI, HDL cholesterol and NT-proBNP.

Abbreviations: ND and D, Non-diabetic and Diabetic group; BMI, Body Mass Index.

### Independent predictors of AD

In order to elucidate the predictive value of each variable on AD level we drew different backward stepwise linear regression models (Table 3). The first model which included age, gender, ACC/AHA clinical stage of HF and the presence of diabetes, showed high predictive strength (R=0.62, p<0.001). In the second model we added NT-proBNP; as the first one, it showed a good predictive strength (R=0.63, p<0.001). In the third and fourth models we introduced BMI first and then HDL cholesterol; the predictive strength of these models was still satisfactory (R=0.62, p<0.001; R=0.69, p<0.001 respectively); however, diabetes lost its independent predictivity.

**Table 3:** Backward stepwise linear regression models

	**Model 1**		**Model 2**		**Model 3**		**Model 4**	
	**R=0.62, <0.001**		**R=0.63, <0.001**		**R=0.63, <0.001**		**R=0.69, <0.001**	
	**B±SE**	**p**	**B±SE**	**p**	**B±SE**	**p**	**B±SE**	**p**
**Age**	0.24±0.05	<0.001	0.17±0.05	<0.001	0.10±0.05	0.021	0.07±0.04	0.124
**Gender**	5.70±1.51	<0.001	4.05±1.49	0.008	4.49±1.41	0.002	1.97±1.47	0.182
**ACC/AHA stage**	2.47±0.60	<0.001	1.77±0.62	0.005	1.38±0.60	0.023	1.40±0.57	0.016
**Diabetes**	−3.44±0.99	0.001	−2.90±0.95	0.003	−1.37±1.02	0.182	−1.86±0.98	0.061
**NT-proBNP**			0.01±0.01	0.001	0.01±0.01	<0.001	0.01±0.01	<0.001
**BMI**					−0.30±0.11	0.008	−0.17±0.12	0.164
**HDL cholesterol**							0.17±0.04	<0.001

Abbreviations: ACC/AHA, American College of Cardiology/American Heart Association; BMI, Body Mass Index.

## Discussion

It is known that plasma levels of AD are able to predict diabetes onset and diabetic patients show lower concentrations of the adipokine compared to the general population [4,6]. Our study extends these observations to a specific subgroup of DM patients with ischemic chronic heart failure, exploring the controversial relationship that exists between AD and HF.

This study confirms our previous observation that AD increases in patients with CAD and overt HF compared to those with normal or reduced LVEF but clinically asymptomatic [19]. This is in accordance to the literature which reports higher AD levels in patients with advanced NYHA class [13]. We now show that not only clinical staging, but also a more pathophysiological classification of HF as proposed by ACC/AHA guidelines, is able to predict AD levels in this subset of patients. NT-proBNP, HDL cholesterol and age still remain the strongest, independent predictors of AD [19].

We have shown that AD levels are systematically lower in diabetics compared to non-diabetics, no matter to what HF staging class they belong. These data suggest that diabetic subjects with reduced LVEF orchestrate a slower rise of AD which begins only in face of overt HF (group C_D_). It is of interest that Chen and coll. in asymptomatic men with uncomplicated type 2 diabetes found a positive association of circulating AD levels with myocardial glucose metabolism but not with cardiac function [30].

Because of its cross-sectional nature, our study cannot elucidate with clarity the pathophysiological mechanisms that lie under the increase of AD at worsening HF. In any case, some considerations can arise from the data. First of all, cardiac cachexia cannot be the main cause of higher AD levels in HF because none of our study subjects was underweight and the increase of AD observed between A/B and C was not coupled to a decrease of BMI. Therefore, cardiac cachexia is a late process in HF compared to the increase of AD which seems to be a faster phenomenon.

For the same reason, we cannot offer a definite explanation for the hampered rise of AD that we detected in HF diabetic subjects. However, we can speculate that diabetics have lower AD plasma levels to begin with which may depend from their higher BMI. Indeed, in our backward stepwise linear regression models, we found that DM loses its predictivity on AD when BMI was introduced as cofactor. However, if we focus on patients with overt HF (C_ND_ vs. C_D_) we find that despite a significant difference in AD levels, BMI was not different (data not shown). These data suggest that something else, beyond body weight, is able to modulate adiponectin metabolism in diabetic patients with HF. A good candidate could be NT-proBNP which seems to directly induce AD production by adipocytes, *in vitro*, and in heart failure patients [17]. Even more striking, natriuretic peptides are able to increase total and HMW-adiponectin concentrations in healthy subjects [31]. It could be assumed that diabetic patients produce lower levels of NT-proBNP in response of overt HF. However, this is not the case: in fact the level of NT-proBNP is not significantly different between diabetics and non-diabetics belonging to the A and B clinical stages. Even more, diabetic subjects in the C group have higher, although not significant, levels of NT-proBNP when compared to the non-diabetic C subgroup, in spite of a lower adiponectinemia (data not shown). A question remains as to what lies between the secretion of NT-proBNP and the production of AD by adipocytes. Some sort of “NT-proBNP resistance” may exist in type 2 DM. Further studies will be required to investigate this hypothesis, comparing the different AD response of diabetic and non-diabetic subjects to injection of natriuretic peptides.

Finally, Won and coll. found that plasma concentration of AD was significantly lower in HF patients with metabolic syndrome compared to HF patients without metabolic syndrome [32]. In our series, the prevalence of metabolic syndrome, as defined by ATPIII criteria [33,34], was 68.2% in diabetic patients and 30.9% in non-diabetic patients. At multivariate analysis, the presence of metabolic syndrome was not an independent predictor of AD plasma levels (data not shown).

### Study limitations

Our population comprises only ischemic HF and type 2 DM. Heart failure of different etiology (valvular, hypertensive and idiopathic) was not explored. Similarly, we have no data regarding type 1 DM. Another limitation of the study is the small number of female subjects (only 13%) that were included which depended on the nature of the inclusion criteria (CAD). Finally, as stated above, all limitations of cross sectional studies also apply to our findings.

## Conclusion

We corroborate the finding that AD raises across the ACC/AHA chronic HF stages but this is less evident in diabetic patients. In type 2 DM the progressive increase of AD in relation to the severity of LV dysfunction is hampered and becomes evident only in overt HF. The different adipokine profile seems to be partially, but not exclusively, determined by the well-known predictors of AD. Other molecular mechanisms, such as a “NT-proBNP resistance”, acting in diabetic patients, can be hypothesized to explain the different behavior of this adipokine.

## Abbreviations

ACC/AHA: American College of Cardiology/American Heart Association; AD: Adiponectin; BMI: Body Mass Index; CAD: Coronary Artery Disease; DM: Diabetes Mellitus; D and ND: Diabetic and Non-Diabetic group; eGFR: Estimated glomerular filtration rate; GDS: Geriatric Depression Scale; Hb: Hemoglobin; HbA1c: Glycated Hb; HF: Heart Failure; IDS: Greenfield’s Index of Disease Severity; LVEF: Left Ventricular Ejection Fraction; 6MWT: 6-min walking test; NYHA: New York Heart Association.

## Competing interest

The Authors declare that they have no competing interests.

## Authors’ contributions

SB and FT conceptualized and designed the study and carried out the analysis and interpretation of data; NM contributed to the design of the study and the interpretation of data; AA, FO, NB, AF, AM, AP, and SS participated to the acquisition and analysis of data; CDS and GL carried out the biochemical analysis including the determination of adiponectin; SF and MM performed the statistical analysis; EM gave an important intellectual contribution by revising the manuscript critically. All authors read and approved the final manuscript.
